# Cultural Implications Regarding Privacy in Digital Contact Tracing Algorithms: Method Development and Empirical Ethics Analysis of a German and a Japanese Approach to Contact Tracing

**DOI:** 10.2196/45112

**Published:** 2023-06-28

**Authors:** Joschka Haltaufderheide, Davide Viero, Dennis Krämer

**Affiliations:** 1 Medical Ethics With Focus on Digitization Joint Faculty of Health Sciences Brandenburg University of Potsdam Potsdam Germany; 2 Faculty of Educational Sciences University of Duisburg-Essen Essen Germany; 3 Faculty of Social Sciences Georg-August-University Göttingen Göttingen Germany

**Keywords:** digital contact tracing, algorithms, methodology, empirical ethics, privacy, culture-sensitive ethics, mobile phone

## Abstract

**Background:**

Digital contact tracing algorithms (DCTAs) have emerged as a means of supporting pandemic containment strategies and protecting populations from the adverse effects of COVID-19. However, the impact of DCTAs on users’ privacy and autonomy has been heavily debated. Although privacy is often viewed as the ability to control access to information, recent approaches consider it as a norm that structures social life. In this regard, cultural factors are crucial in evaluating the appropriateness of information flows in DCTAs. Hence, an important part of ethical evaluations of DCTAs is to develop an understanding of their information flow and their contextual situatedness to be able to adequately evaluate questions about privacy. However, only limited studies and conceptual approaches are currently available in this regard.

**Objective:**

This study aimed to develop a case study methodology to include contextual cultural factors in ethical analysis and present exemplary results of a subsequent analysis of 2 different DCTAs following this approach.

**Methods:**

We conducted a comparative qualitative case study of the algorithm of the Google Apple Exposure Notification Framework as exemplified in the German Corona Warn App and the Japanese approach of Computation of Infection Risk via Confidential Locational Entries (CIRCLE) method. The methodology was based on a postphenomenological perspective, combined with empirical investigations of the technological artifacts within their context of use. An ethics of disclosure approach was used to focus on the social ontologies created by the algorithms and highlight their connection to the question about privacy.

**Results:**

Both algorithms use the idea of representing a social encounter of 2 subjects. These subjects gain significance in terms of risk against the background of a representation of their temporal and spatial properties. However, the comparative analysis reveals 2 major differences. Google Apple Exposure Notification Framework prioritizes temporality over spatiality. In contrast, the representation of spatiality is reduced to distance without any direction or orientation. However, the CIRCLE framework prioritizes spatiality over temporality. These different concepts and prioritizations can be seen to align with important cultural differences in considering basic concepts such as subject, time, and space in Eastern and Western thought.

**Conclusions:**

The differences noted in this study essentially lead to 2 different ethical questions about privacy that are raised against the respective backgrounds. These findings have important implications for the ethical evaluation of DCTAs, suggesting that a culture-sensitive assessment is required to ensure that technologies fit into their context and create less concern regarding their ethical acceptability. Methodologically, our study provides a basis for an intercultural approach to the ethics of disclosure, allowing for cross-cultural dialogue that can overcome mutual implicit biases and blind spots based on cultural differences.

## Introduction

### Background

Digital contact tracing algorithms (DCTAs) and respective apps have emerged as a ubiquitous phenomenon throughout the COVID-19 pandemic [1,2]. They have been advocated as a means to support and improve pandemic containment strategies [3-5], to allow individuals to become involved in attempts to decrease infection rates [5,6], and to protect populations from the adverse effects of COVID-19 [7,8]. A large number of governmental and nongovernmental agencies have implemented DCTAs of different varieties all over the world [9,10]. In most countries, their development and implementation were accompanied by intense public and scientific debates.

From the perspective of health and data ethics, the impact of DCTAs has been discussed, especially with reference to users’ autonomy and privacy, that is, as a potential threat to informational self-determination and a possible step toward (governmental) mass surveillance [11-18]. Privacy in these debates is often understood as the ability to control access to information or the ability to have one’s domain of information [19]. However, recent approaches that understand privacy as a norm that regulates and structures social life have gained increasing importance [15,20-22]. These views stress the social dimension of privacy as a component of well-functioning societies [23]. Privacy in this regard is pluralistic [21], meaning that the question about appropriateness takes into account the contextual situatedness of information flows. *Contextual* can, for example, refer to the societal and cultural backdrop of a situation, way of transmission of information, involved agents, and type of information in question [24-26].

From this perspective, an important part of ethical evaluations of DCTAs would be to develop understanding of cultural factors relevant to their information flows [15,16,27]. For the purpose of this study, we define culture simply as a set of formative conditions imposed on a group of people (eg, through common education, language, shared historical traditions, media, and so on), which is distinct from those conditions imposed on others [28]. Given that DCTAs are a global phenomenon and ethical debates seem to occur in almost all instances but against very different cultural backgrounds, it is remarkable that questions regarding the role and impact of cultural factors within the debate on privacy in DCTAs have almost completely evaded scrutiny so far.

### Objectives

Against this background, this study developed a case study methodology to include contextual cultural factors in the ethical analysis of DCTAs and presented exemplary results of a subsequent analysis of 2 different DCTAs following this approach. Hence, the aim of this study was 2 fold. First, we proposed our methodological approach and second, analyzed the contextual situatedness of 2 DCTAs of different origins regarding their information flow by correlating structures and concepts used in these algorithms with prevalent cultural and societal patterns of their background. We focused on the disclosure of the ontologies created within the algorithms, that is, what concepts and structures are used to represent a social encounter and to screen for risk imposition and the implications of such ontologies when judging the appropriateness of information flows in DCTAs. Therefore, we analyzed 1 algorithm with a Western background and 1 algorithm that stems from Japan. We made this choice because it is known that there are important and significant differences between cultural assumptions, especially regarding the understanding of body, time, space, and technology relevant to the representations of subjects in DCTAs [29,30].

We based our study on a so-called mediating approach of technology [31,32]. Mediating approaches provide a suitable methodological background to study the contextual situatedness of information flows materialized in technologies. In this way, we drew from a phenomenologically oriented and broadened understanding of technology that has become increasingly important in recent years [33-35]. In contrast, our normative perspective was guided by the concept of *ethics of disclosure* [36], that is, ethics as an undertaking to reveal the influence of implicit concepts and values in technological artifacts to allow for their critical reflection from a normative point of view [36-38]. On the basis of these considerations, we will shortly introduce the 2 algorithms—the algorithm of the Google Apple Exposure Notification Framework (GAEFN), as exemplified by the German Corona Warn App (CWA), and the Computation of Infection Risk via Confidential Locational Entries (CIRCLE) algorithm [39-41] from Japan. Both have been advertised as being especially privacy preserving. We have presented the results of our comparative analysis. We showed how both algorithms use the idea of representing a social encounter and how subjects of this encounter gain significance in terms of risk against the background of their temporal and spatial properties, as represented in the calculations. However, the comparative analysis revealed at least 2 major differences. The first one is a different importance of spatial and temporal extensions of the subjects, which is represented in different priorities and algorithmic filter hierarchies. Second, this implies a different condition of existence of the subject itself. With condition of existence, we meant what primarily constitutes a subject and when it *vanishes* from further calculations. Finally, with these results in mind, we have briefly discussed the significance of our analysis for the ethical evaluation of privacy. We have highlighted the implications of our results and the need for further studies and methodological development.

## Methods

### Perspectives About Technology

Our approach was based on 2 premises defining an empirically informed ethical inquiry into technology. The first was a postphenomenological account of technology as a way to guide the perspective toward DCTAs. This account focused on the mediating capacities of technological artifacts. According to Verbeek [42], studying technology in terms of postphenomenology means studying technological artifacts with regard to their relations with human beings. Its focus is on how technologies shape the connection between humans and their lifeworld as mediators of experiences and practices [42]. This perspective was combined with descriptive—often empirical—investigations, that is, the actual technological artifact in its context of use was considered to be the starting point of further inquiry. Consequently, postphenomenological studies depart from a common sense understanding of technology, providing a more complex framework to understand and describe what humans do with technology and what technological artifacts do to them. Understanding technologies as mediators means that artifacts no longer appear as means to an end but are viewed on the basis of their capacity to enter into the phenomenological relation between acting agents and their lifeworld [32,42]. However, the transmission that is occurring with this *relay* is not neutral but is to be understood as a transformation based on the functional capacity of a specific artifact. A simple metaphor to depict this concept is the image of a person wearing eyeglasses. Although these glasses enter into and mediate the visual experience of that person, they do not neutrally transmit an image of the surroundings but change the users’ perception based on the shape of the lenses. Of course, it is implied that the technological artifact itself is a created object and is the result of a process of creation in which implicit and explicit values, cultural assumptions, and societal practices have left their mark. This led to the question regarding how the experience of technology is shaped, and, considering the role of functionality, what implicit concept values or biases may have entered the process of creation and are materialized within an artifact’s functional capabilities.

### Ethics of Disclosure

Answering this question and reflecting on it from a normative perspective is part of what can be called ethics of disclosure [36-38]. Therefore, we based our study on the observation that many technical operations tend to become or are, in principle, opaque, as they include operations, systems, or parts that are very complex and difficult to understand [37,38]. In particular, digital technologies have a tendency to linger at the border of subject and environment and to constantly sink below the phenomenological threshold of perceptibility, either by becoming embodied or by becoming part of the environmental background [43]. Hence, the process of creation and use of digital technology can be described as a constant process of closure in which decisions are made, design is implemented, and properties of the technical object become fixed as a way of producing order [38]. The aim of an ethics of disclosure is to identify such (moral) opacity, to bring it to view, and to reflect about morally relevant or problematic features that can be disclosed through thorough investigation. In other words, the goal is to maintain the reversibility of the many foldings of technology [36].

### Hypotheses for the Investigation

Combining both of these premises led to a focus on an *ontological disclosure*, that is, to ask what kind of *world* we see through a technology (premise 1) and what implicit concepts and standards materialized in a technology influence the shape of this world (premise 2). Regarding DCTAs, we hypothesized that these technologies—in principle—can be described as creating the opportunity to perceive social encounters as health risk imposition and to adapt behavior accordingly. Therefore, DCTAs create (only partially accessible) social ontologies, broadly defined as *what there is* in a certain social horizon, to represent crucial features of a social encounter and to weigh the significance of such encounters in a metric of risk. We further hypothesized that these ontologies also provide valuable insights in the privacy debate as they represent and reconstitute contextual factors that are important when considering the social dimension of privacy.

### Approach to the Analysis

From these hypotheses, we derived the following questions:

1. How is a social encounter represented within the algorithms of DCTAs?
2. How do different representations from different cultural backgrounds relate to prevalent structures and concepts of their context of creation?

To answer these questions, we adopted a comparative case study approach. We defined case studies in accordance with Crowe et al [44] as a methodical approach of in-depth exploration of the complexity and uniqueness of a particular phenomenon [45]. Case study approaches have been widely used in a variety of disciplines and have proven to be especially suitable to explore phenomena in relation to their context and to develop an in-depth understanding. Given our postphenomenological framework and research questions, we understand our approach to be instrumental, that is, the analysis of the cases serves the purpose of understanding a more general phenomenon [46]. In our case, this was the embeddedness of cultural, social, and ethical values in DCTAs. A *comparative* analysis was chosen to create a high-contrast environment for our inquiry that would provide opportunities for substantive insights to emerge.

It is important to understand that this approach was interpretative and exploratory in nature. Interpretative means that we aimed to understand DCTAs not from an objective or external observer perspective but against the background of its creation and use, that is, how a system is understood and is given meaning by a shared understanding of such phenomena [44,47]. As, for example, Ryan et al [48] have put it, it is to focus on the what’s, why’s, and how’s relevant to the ethical questions emerging with technologies. In this way, interpretative approaches aim to explore how meaning is assigned to allow for building and refinement of theories, which may guide further ethical analysis. Consequently, we did not aim to quantify any of our insights (as this would not be appropriate). Rather, first, we presented a material description of our cases. Then, we developed an interpretation based on our material of analysis to finally connect this interpretation to certain prevalent cultural patterns in Western and Eastern cultures.

### Material of the Analysis

Our primary data sources were all publicly available documents and documentation regarding the 2 algorithms from verified sources that were actually involved in research and development. Sources were collected from January 2021 to March 2022, with an update in August 2022. The source collection included scientific publications and presentations, technical documents and manuals, and user documentations. Data were analyzed from a qualitative perspective with the general idea to understand the algorithms and their creation as product and social process of sense and meaning making [45,49]. Hence, our aim was to learn how the creators of the algorithms provide a rationale for their calculations; make sense of their parts; and explain their inner workings, purpose, and functioning. First, we aimed at reconstructing an abstract representation of the information flow of the algorithms as presented in the material and then, to reconstruct meaning and significance that were assigned to different steps of this flow.

### Ethical Considerations

For this analysis, no data about human participants were analyzed, and, hence, we deemed approval through an institutional review board to not be necessary.

## Results

### Material Description of the Cases

**Figure 1:**
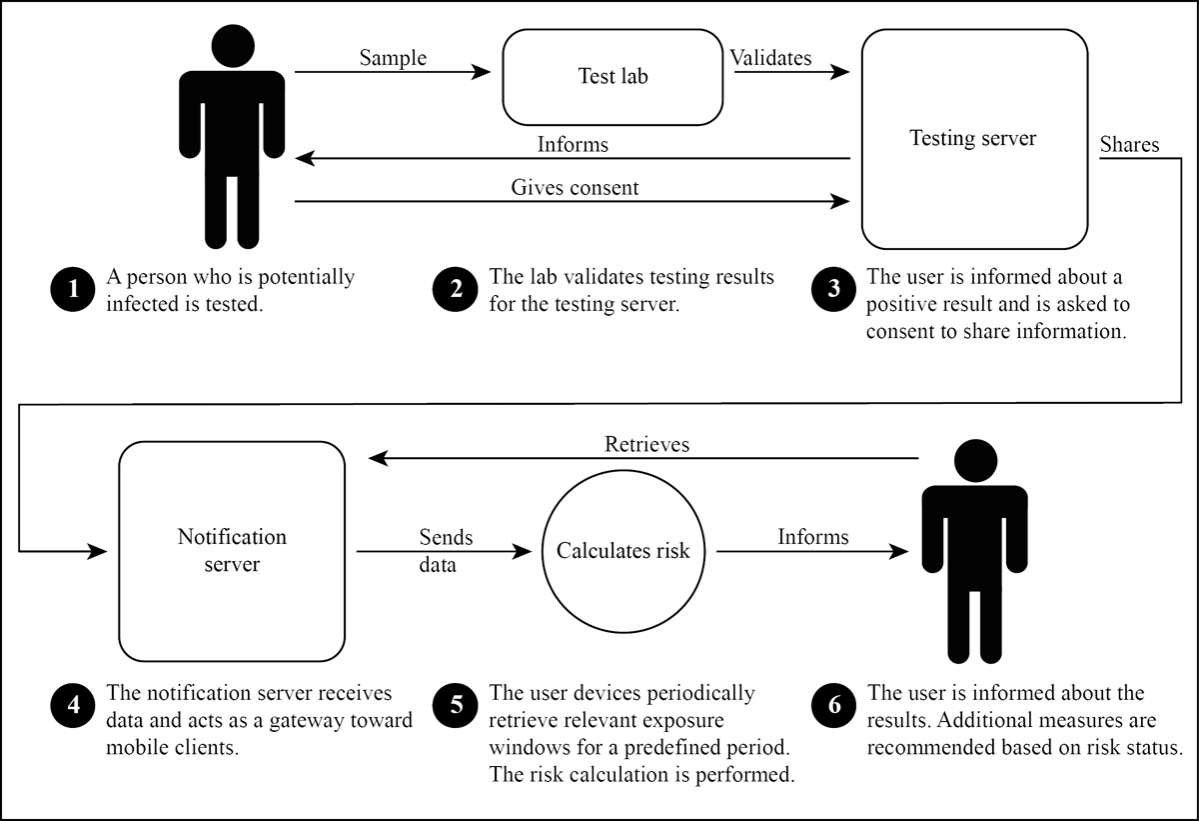
Steps taken within the Google Apple Exposure Notification Framework according to Corona Warn App solution architecture.

Our first case is concerned with the algorithm of the GAEFN in its exemplary implementation in the German CWA. The GAEFN is a prominent framework to implement digital contact tracing and is part of the joint efforts of Google and Apple to create a privacy-preserving contact tracing protocol. It was quickly adopted by many providers of contact tracing apps. Figure 1 provides a general overview of how the GAEFN works in case of the CWA.

The GAEFN allows to easily implement digital contact tracing on smartphones using iPhone Operating System and Android Operating System. It provides a basic architecture to exchange the necessary information between the devices. This includes the following:

1. The ability to gather data and measure properties occurring in case of intersecting Bluetooth cones with other devices
2. The ability to gather data regarding the health status of users
3. The ability to combine both data sources to represent a social encounter between a person who is infected and a person who is potentially affected and to evaluate the health risk of this encounter
4. Auxiliary functions to transmit, store, display, and retrieve data and recommendations

Regarding the first aspect, the GAEFN provides respective apps with information about timing, signal attenuation of the Bluetooth interface, and additional information necessary to identify a contact in case of a later infection. When intersecting Bluetooth signal cones are detected, each device identifies itself with its counterpart with a nonpermanent identifier. This identifier is, then, locally stored for a predefined time together with data indicating the signal damping of the smartphone and the duration of the signal cone overlap.

Regarding the second aspect, the system provides the basis to collect infection status (positive testing result) and data indicating the risk of transmitting the virus for each day in a predefined time frame. The latter value can be derived from a variety of different mathematical functions, which basically aim to describe the most likely course of illness with reference to the carriers’ infectiousness over time. The third aspect provides an algorithm to match recorded identifiers with a list of positively flagged identifiers by the second aspect and then, to evaluate whether and to what extent this encounter can be described as a health risk imposition based on its properties. Precisely, the third aspect is the primary target of our analysis. The evaluation can be displayed as the fourth aspect to the user to inform them about their potentially harmful contact and their overall risk and to recommend further measures.

**Figure 2:**
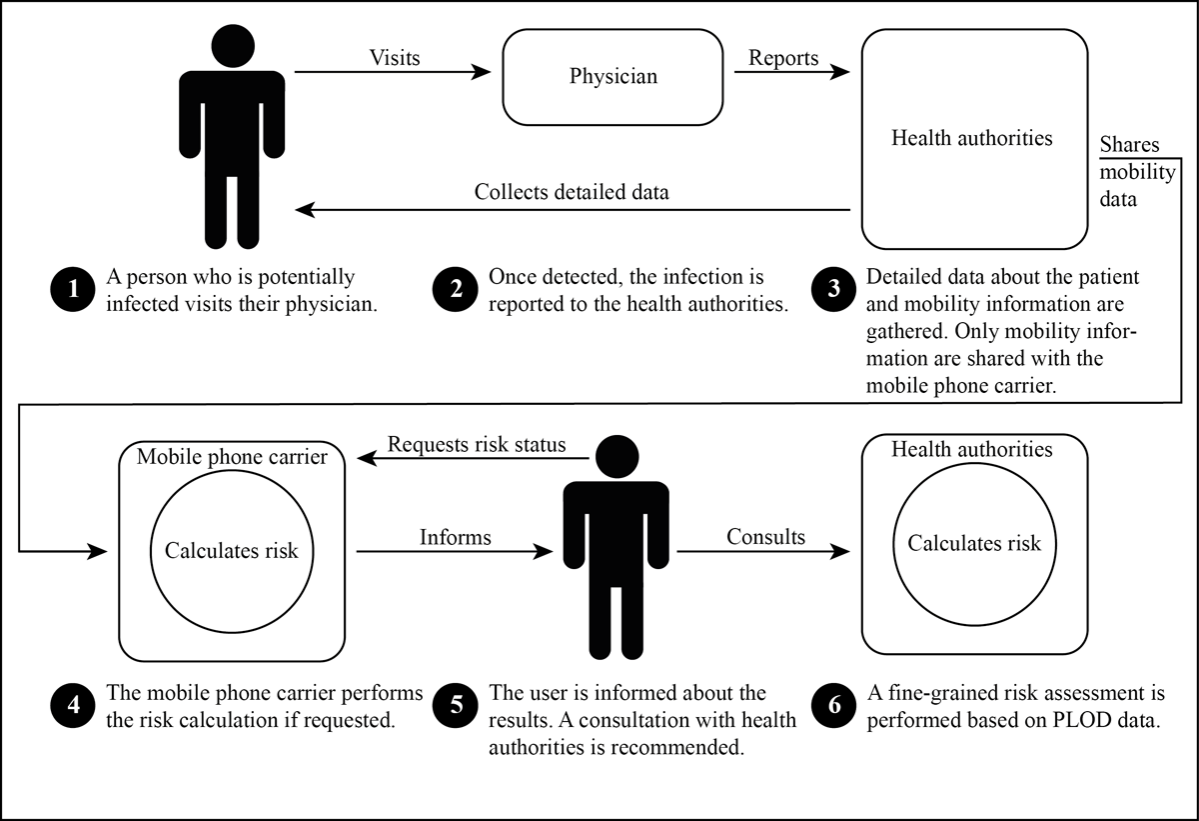
Steps taken with the Computation of Infection Risk via Confidential Locational Entries method according to Ami et al [39]. PLOD: Patients’ Locational Open Data.

The second case is the so-called CIRCLE method. In many Asian countries such as Japan, South Korea, or Taiwan, differing approaches for DCTAs have been discussed and tested as early as 2010 [39]. The CIRCLE method is one of the young offspring of this family of approaches. Figure 2 provides an overview.

The CIRCLE method includes the following:

1. Gathering infection status and locational information about a person who is infected
2. Gathering locational information about users of the mobile cell grid
3. To combine both of these data and to evaluate the likelihood of a contact between the person who is infected and any other person within the cell grid
4. Auxiliary functions and methods to communicate with health authorities and users

When a person is confirmed to have tested positive for a contagious disease such as COVID-19, the consulted physician reports the incident to the health authorities (first aspect). These authorities then gather additional data and share the patients’ mobility information with the operators of the mobile phone grid under a nondisclosure agreement. Mobile phone owners can request a clarification of potentially risky encounters or allow clarification and information in advance. Mobile operators will gather the locational information about the requesting user (second aspect) and will evaluate the risk of a contact. On the basis of the outcome of the mobile operator’s inquiry, the probability of contact can be calculated and provided to the potential contact person to recommend further follow-up with the health authorities and a fine-grained risk assessment using a standardized data structure called Patients’ Locational Open Data (PLOD). PLOD provides manually gathered locational information in a standardized form and, hence, allows an assessment of the encounter based on the names of places visited or exact time of stay.

### Social Ontologies of GAEFN and CIRCLE

**Figure 3:**
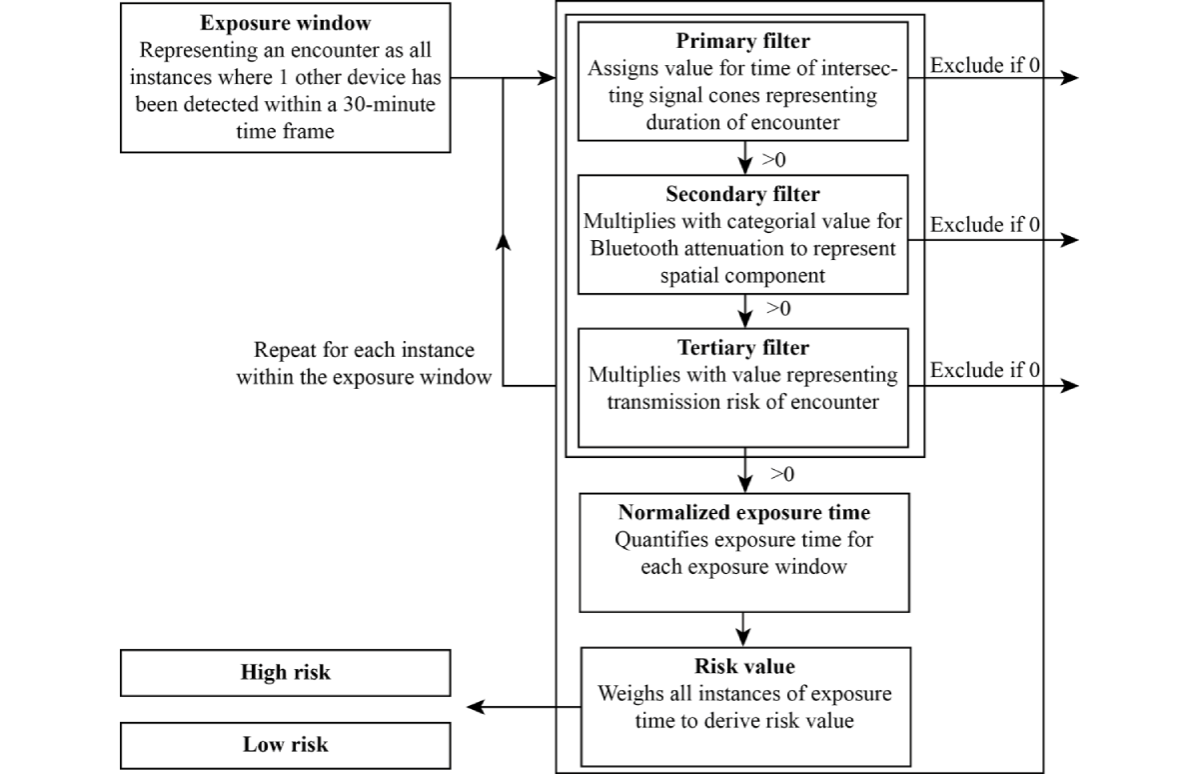
Evaluating social encounters as health risks in the Corona Warn App.

**Figure 4:**
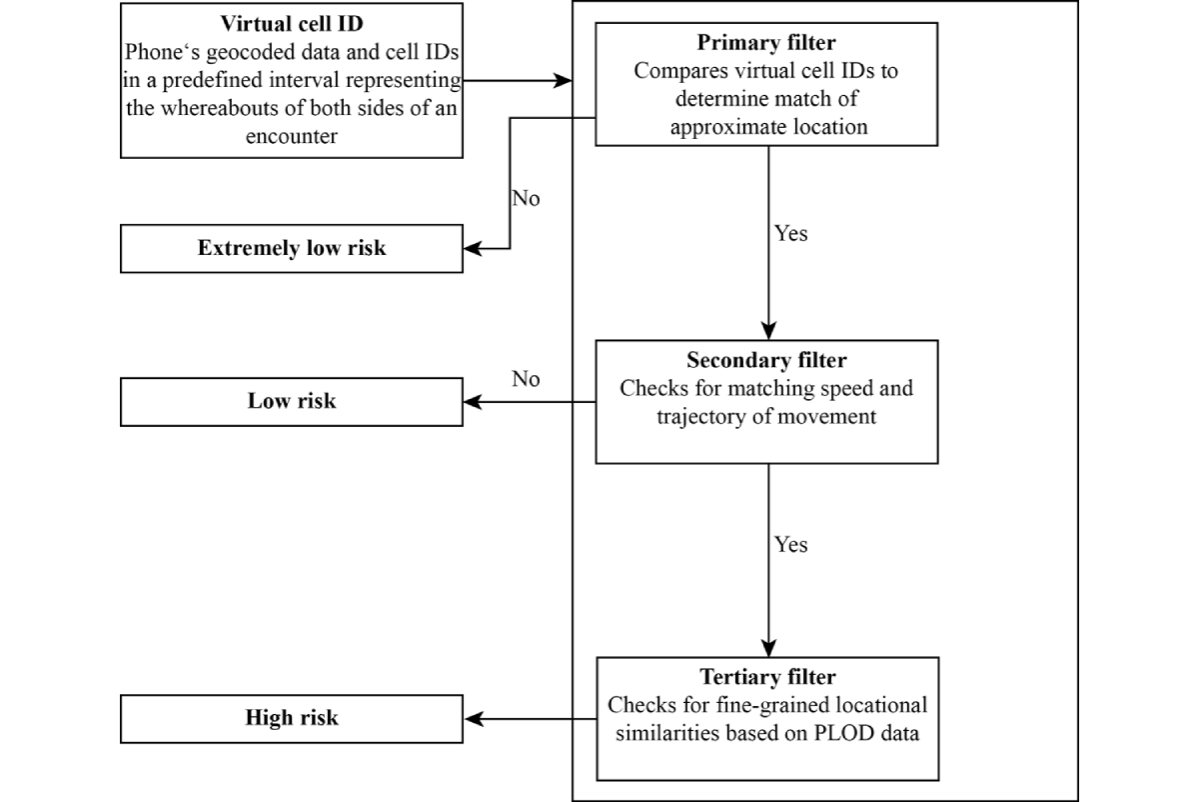
Evaluating social encounters as health risks in Computation of Infection Risk via Confidential Locational Entries. PLOD: Patients’ Locational Open Data.

Figures 3 and 4 show the abstract representations of the information flow of GAEFN and CIRCLE based on our analysis. The GAEFN algorithm is described as being based on the so-called exposure windows, which are provided through the framework to perform a quantitative numerical assessment of an encounter. An exposure window is any intersection with any other Bluetooth cone determined by matching identifiers within the system within consecutive 30-minute intervals. An exposure window represents an encounter to evaluate a risk exposure. The CWA solution architecture documentation, for example, states the following: “All exposure events are collected by the ENF internally and are split up into ‘Exposure Windows,’ which represent all instances where one other specific device (without known identity) has been detected within a 30 minute window” [50].

For every exposure window within a predefined period, the algorithm performs a risk assessment. This algorithm comprises 3 evaluative stages and a final cumulative stage. In a primary estimation, every recorded match is assessed for the duration of intersecting Bluetooth cones representing the duration of the encounter. If the duration is below a predefined threshold, the encounter is effectively excluded from any further consideration. Technically, this is done by setting a multiplier of the calculation to 0, rendering any further calculation ineffective, as the numerical value from now on will always be 0. If a non-0 value can be assigned, this basic value is then weighed (multiplied) with a value derived from the Bluetooth antenna attenuation as a proxy measure for distance. The attenuation estimator represents a component of spatial distance within the algorithm. It is established as forming “four attenuation ranges (sometimes also called ‘buckets’), which have a specific weight applied” [50].

Bucket border correspondence with certain distances is described as being experimentally established as *ground truth*. Again, depending on the predefined parameters, a multiplier is set to a value representing the distance between both sides of the encounter. Values above the threshold indicating great distance will set the multiplier to 0. In any other case, the derived value will finally be multiplied with a quantifier representing the transmission risk, that is, the infectiousness estimated over time. The calculation results in what is called a *normalized exposure time* representing the time under risk in each exposure window. In the last stage, exposure windows can be cumulated to derive a total risk score, which is fed back to the user as categorical value (high, low, or undetermined).

In contrast, regarding the CIRCLE algorithm, the unit to represent an encounter is derived from so-called virtual cell IDs. A virtual cell ID comprises geocoded locational data based on the mobile cell grid, which is compressed into a 2D array representing locations in predefined intervals. Hence, a virtual cell ID represents the spatial location of a person at a given time based on the mobile phone grid. The algorithm is comprised of 3 filter stages designed to sort out potential contacts. The primary filter evaluates by comparison, whether the cell IDs of 2 users match at any time to determine any likelihood of an encounter, that is, the meaningful possibility that both sides could have been at the same location. If this is not the case, the risk is considered to be extremely low and the potential contact is excluded from further calculation.

The second filter evaluates whether the person who is infected and the person who is potentially affected can be considered to be (1) moving (2) at a similar speed and (3) on a parallel pathway. First, an approximation of the moving speed of the requesting user is estimated to exclude all individuals who can be seen to move on foot and therefore will be potentially outside in the open. For these encounters, risk is considered to be very low. They are, hence, discarded. For all others, a so-called Jaquard coefficient is calculated to represent the similarity of the users’ movements through the mobile cell grid. If a comparative analysis reveals sufficient similarity, the person who is infected and the user are considered to be using the same transportation system at the same time and hence might have come into close contact. On the basis of this step, representing a *probable contact* with people who are potentially affected can be informed and further followed up with the health authorities, that is, ideally performing the last suggested step of the algorithm. If triggered by the user, health authorities will gather additional data from the person who is potentially affected and include it in the system to confirm locational overlaps at a fine-grained level using the so-called PLOD data.

## Discussion

### Principal Findings

Our analysis shows that both algorithms use the idea of representing a social encounter of 2 subjects. These subjects are coming into existence and gain significance in terms of risk against the background of their temporal and spatial properties, that is, by a representation of the temporal and spatial extension of human existence. However, by taking a close look, the comparative analysis reveals at least 2 major differences. The first one is an obviously different importance of spatial and temporal extensions of the subjects in an encounter, which is represented in different priorities and filter hierarchies. Second, this implies a different condition of existence of the subject itself. With condition of existence, we mean what primarily constitutes a subject and when it vanishes from further calculations.

Regarding the first aspect, the GAEFN clearly prioritizes temporality over spatiality. Given that the calculations of the GAEFN are based on what is called an *exposure window* and subjects effectively vanish from the calculations when certain thresholds in duration are not met, one may want to say that a subject as represented in the encounter only comes into existence against a temporal background and its significance in terms of risk is primarily based on evaluating its diachronic dimension. In contrast, the representation of spatiality is reduced to mere distance without any direction or orientation. However, the CIRCLE framework prioritizes spatiality over temporality. The calculation is based on locational data, that is, subjects come into existence by occupying a spatial position against the background of a Cartesian coordinate system based on the mobile phone grid. The significance in terms of risk is determined based on the spatial location relative to the others, either in terms of matching cell IDs or synchronous movement. Hence, although subjects within the GAEFN are essentially represented based on their temporal properties, that is, being existent over a certain time, CIRCLE is based on a representation of subjects in terms of spatial properties, namely, by being in certain places or by being in a spatial relation with certain others.

To our understanding, these prioritizations can be seen to align with important cultural differences in considering basic concepts such as subject, time, and space in Eastern and Western thought. As a way to illustrate these differences in more detail, it is insightful to turn to the respective traditions of thought as exemplified in continental and Japanese philosophy. In the following sections, we refer to these traditions as a way to illustrate how the differences we found in our analysis exemplify certain cultural patterns that can also be found in these traditions.

The priority of temporality over spatiality is paradigmatic for Western thought and can be traced back to influential thinkers, such as Aristotle, Augustinus, Descartes, and Kant. Probably, the most influential analysis in the 20th century has been conducted by Martin Heidegger. His thought on time, space, and self might be particularly insightful for 2 reasons. First, Heidegger aims to outline how one may conceive oneself as being related to time and space—or, to put it simple, how one may find themself represented as temporal and spatial subject in their thinking. Second, Heidegger’s thought has sparked extensive criticism from Japanese philosophers, leading distinguished scholars to clarify Japanese concepts and to point out differences [51,52].

For Heidegger, temporality provides the basis of every understanding of the self (Dasein) and what it means to be in the world [29,53]. On the basis of an understanding of the self as activity of being in the world, Heidegger describes it as an existential structure that solely exists in the activity of encountering the world [54]. Temporality, perceived as past, present, and future, in turn, provides the basis for the coherence and connectedness of the experiences of the self. Thus, temporality is the constitutive horizon and condition of any existence. It provides a horizon to every understanding and interpretation of being in the world. In contrast, spatiality plays a subordinate role [51,55]. According to the Heideggerian account, the body and its spatial extension are not part of the primary existential structure of the self but are derivatives. Therefore, Heidegger’s notion of self, time, and space starts from the idea of the self as an individualistic encounter with a world, understanding temporality as a condition of existence. Hence, the concept favors the individual over the social aspects of human existence and the temporal over the spatial—a prioritization clearly correlating with the analysis of the GAEFN, as outlined previously.

In contrast, Japanese scholars have extensively criticized the concepts proposed by Heidegger and others [52,56,57]. They have, for the most part, rejected the Western ideas for being very individualistic and missing the essence of human existence, especially regarding its social and spatial nature. In doing so, their arguments can be traced back to an understanding of space, time, and self-rooting in a different linguistic tradition and the influence of Buddhist thought.

For example, it has been pointed out that in the Japanese language, the character 間, usually read as *ma*, which, owing to its broad use in different contexts, is typically the most used character to refer to the concept of *space*. According to Kodama [58], *ma* denotes a holistic relationship capable of connecting the continuity and discontinuity of disparate matters and events. It is to be understood as a spatial gap or an opening from which all other phenomena emerge into the flow of dynamic existence. With this significance, space occupies a position similar to Heidegger’s temporality, providing an existential condition of existence. In addition, when observing the word for human being (*ningen*), the first character *nin* (人) means person, whereas the second is spatial and is the same character previously introduced as *ma* (間).

Adding to this background, modern philosophers in Japan strongly shaped this discussion with their views about space and the self and have highlighted the importance of spatiality in Japanese culture. Nishida Kitarō, one the most influential philosophers of modern Japan, for example, developed what he calls a logic of place upon the idea of *absolute nothingness* (*zettai mu;* 絶対無), which stands in sharp contrast with the view of Western philosophers [59].

Nishida builds on the idea of so-called *pure existence* as the primary substratum of the self and *absolute nothingness* [60]. According to Nishida, the basic form of human existence is not the existence of an individual equipped with sensory and mental abilities who engages with an outside world. Rather, in its purest sense, existence precedes the difference between subject and objects. The self as a subject that has self-awareness emerges only in interaction and opposition with what they perceive [61]. Put simply, a person thinking is constituted through the opposition to the object they think about. However, to be and to exist in this sense means being situated spatiality. Hence, the true individual, according to Nishida, is spatial in the sense of standing in opposition to other individuals and objects.

Other influential philosophers of the 20th century have extensively built on this basis. A well-known example would be Watsuji Tetsurō, who became known as Japan’s premier ethical theorist and historian of ethics [62]. Watsuji does not distinguish between the concept of space and the live world (as did Nishida with pure existence and the emerging self-aware subject) but refers to the latter directly. On the basis of a sharp criticism of Western concepts of the self and consistent with the Buddhist tradition, Watsuji stresses that the structure of existence is no less spatial than temporal and no less social than individual [51,57]. When considering the concept of the human self, spatiality cannot be subordinated to temporality nor can the social nature be subordinated to the individual nature of human existence. Hence, Watsuji develops a concept based on the idea of a social-individual duality of being based on the concept of betweenness [51,63]. To exist means to be related to others, whereas being related does not refer to the connection of 2 atomistic individuals but describes the constitution of the subject itself. In contrast, spatiality is the phenomenological concretion of this betweenness, and because human beings’ existence *is* this betweenness, their self is spatial and being in the world will always express itself spatially [57].

More recent examples of Japanese philosophers can be seen to continue on this path, making it possible to identify a thin red line connecting the conception of the self with its surroundings, thus presenting the impossibility of removing the human being from the spatial context in which it lives. In contrast to the ideas exemplified by Heideggerian thinking, this shows a highly spatial identification of the self. One’s identity is linguistically and conceptually fixed in spatial or locational terms and related to or merged with the object world. This view, also referred to as *lococentrism* [64,65], is echoed in the analysis of the CIRCLE method.

### Evaluating the Appropriateness of Information Flows

Regarding the concept of privacy and its ethical evaluation, our analysis suggests important implications. As outlined previously, we understand privacy based on its social value as the appropriate flow of information against a specific background. With this, we refer to a family of approaches that at least partly rejects a classical understanding of privacy in its literal sense. This rather common sense idea of privacy is based on autonomy as noninterference, resulting in a right to have one’s own informational sphere separated from the public or to be in control of who has access to certain information [19,66]. In contrast, social accounts of privacy argue that especially digital large-scale technologies (eg, platform technologies, social media networks, and others) do not primarily endanger individual interests but may influence society and social life to a more general extent [19]. Therefore, it has been argued that privacy has a social value as a norm that regulates and structures well-functioning societies through protection of individuals [23]. Hence, it is the ability of individuals to be properly embedded in differing and different social relations in the sense of being able to have social relations based on different grades of information and in different social roles and identities at the same time [22]. This makes privacy an essentially pluralistic concept. The appropriateness of a privacy norm has to be judged relative to the context of information flow. Nissenbaum [67] has extensively elaborated on the meaning of context, defining it as structured social setting with characteristics evolving over time, which is subject to a host of contingencies. This includes the influence of purpose, place, culture, historical accident, and so on. Consistent with Nissenbaum [67], our study shows that such contingencies are at least partly represented in the different DCTAs ontologies of time, space, and subject.

With this in mind, our interpretation of subject, temporality, and spatiality represented in the algorithms point to 2 very different contexts. Although the GAEFN favors temporality over spatiality following the Western tradition of a self, emerging against the background of temporality as a constitutive condition, the CIRCLE algorithm echoes the lococentrism exemplified by the abovementioned Japanese scholars and traditions. In this regard, CIRCLE follows the prioritization of spatiality as a constitutive condition situating the subject as related to a spatial coordinate system and other individuals.

On the basis of the study by Capurro [68], who has investigated the different ideas of spatiality and temporality and their roles as contextual factors in privacy debates, we suggest that these differences essentially lead to *2 different* normative questions about privacy that are raised against the respective backgrounds. The privacy question emerging with the GAEFN is based on an understanding of the self as a diachronic, continuous entity. Accordingly, protecting the ability to be embedded in a variety of different and dissimilar social relations in view of a continuous temporal self becomes key [68]. In contrast, the privacy question against the background of the CIRCLE algorithm revolves around a spatial and social situatedness of the self that emerges out of its in-betweenness. From an ethical perspective, evaluating privacy in this context would call for a set of rules protecting the ability to be in distinct social relations based on different spatial locations.

### Implications for Ethically Evaluating Digital Contact Tracing

Drawing on these results, 2 important implications for the ethical evaluation of DCTAs need to be highlighted. First, we suggest that the ethical evaluation of privacy in DCTAs needs to be viewed against its respective social and cultural background and the specific technology that raised the question. Since the beginning of the pandemic, different approaches to contact tracing from different contexts have been discussed comparatively. Our analysis shows that this discussion can be highly problematic if it is not aware of the different cultural backgrounds and thus ignores the context for the question about the appropriateness of information flows. What might be a suitable implementation of privacy-preserving measures in one context does not need to be appropriate in another. More specifically, our results suggest that different measures to design and implement privacy-preserving DCTAs can be recommended. However, this may not be important for current technologies alone. Although the pandemic may be about to end, the global impact of COVID-19 has raised awareness about the interconnectedness of global problems and about the vulnerability of health care systems in catastrophic events. Pandemic preparedness has received increasing attention as an important field of interdisciplinary research aiming to systematically improve conditions for a resilient and efficient reaction in case of future emergencies. It is highly likely that any strategic preparation for the future will include plans for the use of DCTAs or similar technologies built upon current knowledge [69]. Recent assessments of strategies have shown that successful national approaches include knowledge about broad contextual factors relevant to decision-making in crisis such as knowledge about national or regional differences, societal values, culture, and ethics [70,71]. A culture-sensitive assessment of DCTAs, as is suggested by our exemplary analysis may, hence, help to gather important information for the development of future strategies or to develop technologies for the future that fit more easily in their context and create less hesitancy in user uptake and less concerns about their ethical acceptability.

### Methodological Implications

From a methodological perspective, our approach to the analysis of DCTAs shows a certain fruitfulness that, to our understanding, should be used further. On the basis of a specific understanding of technology and the aim of disclosing concepts and structures within technological artifacts as an important part of ethical reflection, our methodology provides the first basis of what one may want to call an intercultural approach to the ethics of disclosure in a more general sense [68]. Connecting the insights that can be gained through such an analysis to the debate about privacy in digital contact tracing illustrates how this method can be used to gain in-depth understanding and to instruct ethical debates from a cultural perspective. As Capurro [68] has rightfully argued, this does not only allow to compare similarities and dissimilarities of concepts and structures but also to develop debates toward a cross-cultural conceptual or even moral consensus. Most importantly, it allows to engage in a cross-cultural dialogue that is able to overcome mutual implicit biases and blind spots based on a more nuanced understanding [68].

### Limitations

However, at this point, we have to concede that the approach has limitations that should be addressed by future studies. In contrast, our method represents a new approach in the analysis of technologies that have hardly been used so far. Its acceptance will essentially depend on its validity and further development. We have presented its main premises in the context of this study. Nevertheless, we cannot present a comprehensive approach in full detail but only a first draft. Furthermore, regarding our empirical analysis, limitations have to be noted concerning the choice of material and methods. As outlined previously, both were chosen for the sake of a high-contrast analysis. Hence, our results may be of limited generalizability and transferability in respect to other DCTAs. However, we did not aim for reaching generalizable and transferable results but rather at developing a generalizable method that could be applied under different circumstances to provide valuable insights.

### Conclusions

Bearing these limitations in mind, we have described—to the best of our knowledge—the first culturally sensitive ethics analysis of privacy issues in digital contact tracing. To this end, we have proposed a method based on a postphenomenological understanding of technology, the idea of ethics of disclosure, and a concept of privacy focusing on its social dimension. By relying on a case-based analysis of 2 different contact tracing algorithms, we have argued for essential divergences of these algorithms rooted in different cultural patterns of understanding the spatial and temporal situatedness of the self.

## Abbreviations

CIRCLE: Computation of Infection Risk via Confidential Locational Entries
CWA: Corona Warn App
DCTA: digital contact tracing algorithm
GAEFN: Google Apple Exposure Notification Framework
PLOD: Patients’ Locational Open Data

## Data Availability

The data sets generated and analyzed during this study are available from the corresponding author upon reasonable request.
